# Diagnostic Performance of Six Rapid Antigen Tests for SARS-CoV-2

**DOI:** 10.1128/spectrum.02351-21

**Published:** 2022-03-16

**Authors:** Jessica Navero-Castillejos, Climent Casals-Pascual, Sofía Narváez, Genoveva Cuesta, Juan Carlos Hurtado, Mariana Fernandez, Mireia Navarro, Aida Peiró-Mestres, Ma Victoria Lasheras, Patricia Rodriguez, Andrea Pulgarín, Ma Ángeles Marcos, Jordi Vila, Miguel Julián Martínez

**Affiliations:** a Department of Clinical Microbiology, Hospital Clínic de Barcelona, Barcelona, Spain; b Barcelona Institute for Global Health (ISGlobal), Hospital Clínic de Barcelona, Universitat de Barcelona, Barcelona, Spain; Johns Hopkins Hospital

**Keywords:** SARS-CoV-2, rapid antigen test, diagnosis

## Abstract

Microbiological diagnosis of severe acute respiratory syndrome coronavirus 2 (SARS-CoV-2) has been a challenge. Although real-time reverse transcription PCR (RT-PCR) represents the gold standard method, strategies that allow rapid and simple diagnosis are necessary for the early identification of cases. In this study, we evaluated the diagnostic performance of six different commercial rapid antigen tests (Coronavirus antigen [Ag] rapid test cassette [Healgen Scientific, Houston, TX, USA], COVID-19 Ag FIA [Vircell, SD Biosensor Inc., Gyeonggi-do, Republic of Korea], Clinitest rapid COVID-19 antigen test [Siemens, Healthineers, Erlangen, Germany], SARS-CoV-2 rapid antigen test [SD Biosensor; Roche Diagnostics, Basel, Switzerland], Panbio COVID-19 Ag rapid test device [Abbott, Chicago, IL, USA], and SARS-CoV-2 test [MonLab, Barcelona, Spain]) in 130 nasopharyngeal swab samples tested previously by RT-PCR. The overall sensitivity of the rapid tests ranged from 65% to 79%, and the specificity was 100% for all of them. The sensitivity was higher for those samples with RT-PCR cycle threshold (*C_T_*) values below 25 and those from patients presenting within the first week of symptoms. The Siemens test showed the highest sensitivity for patients with high viral loads while the Vircell test performed better than the rest for *C_T_* values of ≥25.

**IMPORTANCE** The rapid detection of people infected with SARS-CoV-2 is essential for a correct and effective control of the disease it causes. This process must be sensitive, fast, and simple, and it must be possible to carry out in any type of health center. Rapid antigen tests are the answer to this need. Knowing its ability to detect the virus in different stages of the disease is essential for a correct diagnosis, which is why this study has been carried out to evaluate the sensitivity and specificity of 6 different antigens tests in nasopharyngeal smear samples.

## INTRODUCTION

The severe acute respiratory syndrome coronavirus 2 (SARS-CoV-2) pandemic has been a challenge not only for infection surveillance but also for diagnosis. Since December 2020, it has caused more than 258 million cases and over 5.17 million deaths worldwide (1). Although vaccination is greatly effective, the rapid identification of cases and patient isolation remain crucial strategies in terms of infection control. In this context, real-time reverse transcription-PCR (RT-PCR) represents the gold standard diagnostic method for SARS-CoV-2 infections. However, RT-PCR assays are time consuming, are expensive, and require trained personnel. Thus, this type of diagnosis is not feasible in some health centers (2, 3). In such cases, simple and rapid strategies for SARS-CoV-2 detection, like antigen-detecting rapid diagnostic tests, offer a suitable alternative for the early diagnosis and control of the infection. The direct qualitative detection of the viral nucleocapsid protein in nasopharyngeal swabs can be performed using lateral flow tests (LFTs) with a turnaround times of 15 to 30 min. Several of these LFTs currently in use are based in immunochromatography and have marked a turning point in the diagnosis and control of the pandemic (4). The objective of this study was to evaluate the diagnostic performance of six rapid antigen tests (RATs) for SARS-CoV-2 detection compared with RT-PCR, using nasopharyngeal swabs samples in viral transport medium (VTM).

## RESULTS

### Sensitivity (S) and specificity (Sp) of the rapid antigen tests.

Fifty-eight out of the 80 positive samples (72.5%) showed an RT-PCR *C_T_* value of <25, and 42 samples (52.5%) were collected during the first week of symptoms. All six RATs were performed in all samples tested by RT-PCR.

**FIG 1:**
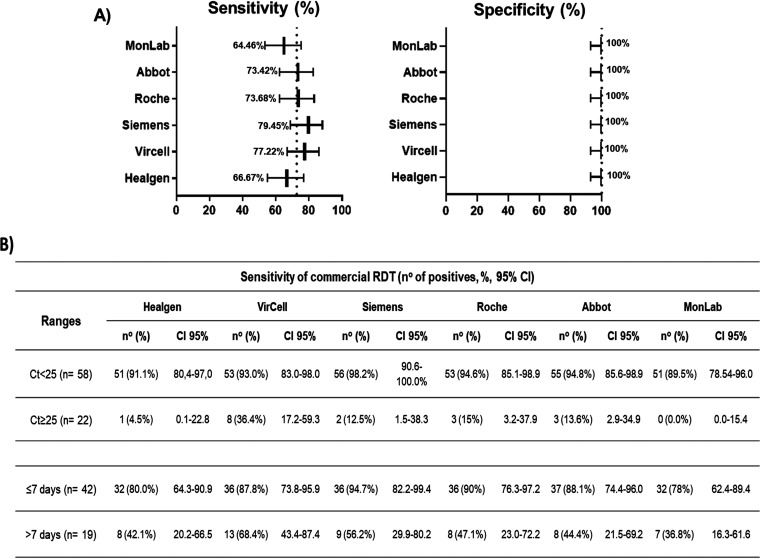
Diagnostic performance of the rapid antigen tests evaluated in this study. (A) The overall sensitivity and specificity obtained for each test are represented in the forest plots. The dotted line indicates the mean of the sensitivity and specificity of all rapid antigen test evaluated. (B) Sensitivity was also calculated in ranges of *C_T_* (<25 and ≥25) and days of onset (≤7 and >7). All values were calculated with a confidence interval (CI) of 95%.

The overall sensitivity and specificity of the RATs evaluated are shown in Fig. 1A. Sensitivity values ranged from 64.5% (MonLab) to 79.4% (Siemens). Four out of the six RATs showed sensitivities over 70%. No false-positive results were detected with any of the RATs, and therefore, specificity was 100% for all tests.

The RATs were more effective in samples with a high viral load (low cycle threshold [*C_T_*] values) than in samples with a high *C_T_* value (low viral load). Figure 1B shows the sensitivity of the RATs when samples were divided according to the *C_T_* value. When *C_T_* values were <25, the sensitivities were close to, or higher, than 90%, whereas in samples with *C_T_* of ≥25, the sensitivity decreased notably. Most kits did not detect more than 15% of the samples with *C_T_* values of ≥25. The Siemens RAT showed the highest sensitivity for high viral load samples (98%), whereas the Vircell RAT was the test detecting more samples with *C_T_* values of ≥25 (36%).

The sensitivity of the RATs fluctuated according to the days of the onset of the symptoms (Fig. 1B). The sensitivity in samples with ≤7 days of symptomatology was around 88%, and the Siemens test showed the best results (95%). However, when the days of the onset were >7, the sensitivities were reduced drastically to values between 37% (MonLab) and 68% (Vircell). Only 7 samples were obtained from asymptomatic patients and 12 from patients without any information about the onset of symptoms. In these cases, they were better detected when the *C_T_* values were lower than 25 (data not shown).

### Negative predictive value and positive predictive value.

As the samples selected had a high percentage of positive results (62%), we calculated the posttest positive and negative predictive values (PPVs and NPVs, respectively) at different disease prevalences that were representative values of those reported during the pandemic in our setting, namely, 1%, 5%, 10%, 15%, and 20% (Table S1). Because of the high specificity presented by the antigen tests in this work, the PPV of all of them was 100% under all the conditions evaluated.

The NPV varied according to the sensitivity presented by the test and the disease prevalence. Nevertheless, although in a prevalence of the 20%, the NPV was lower than that in the other situations, it was still higher than 90%. With a disease prevalence of less than 10%, the NPV showed by the different kits exceeded the 95%, approaching 100% in the case of the 1%. The accuracy for the different prevalence values showed a value greater than 90% in all the kits evaluated. The tests with the best values in terms of PPV, NPV, and accuracy were those of Siemens and Vircell, according to their higher sensitivities.

## DISCUSSION

This study compared the effectiveness of 6 rapid antigen tests, 1 of them automatic, to detect SARS-CoV-2 infections. This rapid screening procedure is now used widely in health centers to screen incoming tourists or to grant access to a wide array of cultural events, where there is a significant risk of virus transmission.

In previous studies, rapid antigen tests have presented a great sensitivity regardless of the symptomatology, and they were found to be more effective in patients with a high viral load (*C_T_* of < 25) (5–9). Under these conditions, the sensitivity of the evaluated tests in this work was also more than 90% (except in MonLab assay, which was 89.5%) and the specificity was 100%. The best results were observed with the Siemens assay (98.2%). While these values correspond with the same ones obtained by Merino-Amador et al. (9), they found a higher sensitivity in patients with a *C_T_* of >25 (28.1% versus 13% in our case). Several studies have also evaluated the Panbio COVID-19 antigen (Ag) rapid test obtaining sensitivities ranging between 80% to 90% which increased above 95% when the *C_T_* value was lower than 25 (7, 10, 11). Although we observed a slightly low overall sensitivity (70%) like that obtained by Andreani et al. (12), this finding was in line with our results. Despite that high sensitivities of the Roche SARS-CoV-2 rapid antigen test were first described in samples with a *C_T_* of <30 (13), the results obtained in our comparison were more similar with those observed recently (i.e., 95% to 100% only for *C_T_* values of <25) (5, 14). Another study by Favresse et al. reported for the first time the effectiveness of the Healgen rapid test for SARS-CoV-2 diagnosis (5). In their work, they observed a sensitivity around 76% which increased to 96.6% in samples with a *C_T_* under 25. These results were supported by later studies that also indicated a lower specificity with respect to other antigen tests (80% to 88%) (10, 15, 16). Even though in our study this test showed a lower sensitivity (66.7% and 91%, respectively), the specificity was 100% in all cases. Of note, our study is the first to evaluate the MonLab tests. When the viral load decreased with *C_T_* values above 25, no test presented a sensitivity greater than 15% except the Vircell automatic reader, which detected 36.4% of the samples with *C_T_* values of ≥25. The automatic reading of the fluorescent signal can be more sensitive than visual inspection of some of the RATs, which may account for the higher sensitivity of the Vircell assay for samples with a low viral load (17, 18).

Currently, there is no consensus on the influence of the presence or absence of symptoms on the sensitivity of the antigen tests. While several studies show that the sensitivity of RATs decreases in asymptomatic cases (8, 13, 19, 20), it is unclear if it is related to the viral load, since other studies have reported that the viral load might be similar in symptomatic and asymptomatic infections (21, 22).

However, it has been reported that in symptomatic patients, the viral load tends to decline over time (23, 24). In our study, we observed that in samples collected within the first week of the onset of clinical symptoms, the sensitivity ranged from 79% to 95% but decreased to 45% when the sampling took place after 7 days, except for the Vircell antigen test (68%). This finding has been reported previously in the literature (6, 9, 11, 17, 25).

Despite the low effectiveness presented by the RATs in patients with more than 7 days of symptoms or low viral load, the high sensitivity shown in acute infections with significant viral loads (*C_T_* of < 25 or ≤7 days of onset) makes them a good tool to identify highly infectious patients. This result is relevant in terms of infection control, given that it has been reported that a small proportion of patients with very high viral loads might be responsible for the transmission of a great proportion of SARS-CoV-2 infections (26–28).

## MATERIALS AND METHODS

### Patients and sample selection.

The study was conducted between November 2020 and January 2021. The specimens were nasopharyngeal swabs (NPSs) collected in 3 mL of VTM (Quimigen, Spain) from patients with suspected COVID-19 infections or from people who had been in contact with confirmed cases. All patients were attended in our hospital or associated primary health centers. We selected a panel of 130 samples tested previously by standard RT-PCR methods; 80 samples were positive and 50 were negative. All 130 samples corresponded to individual patients and were stored at −80°C until testing. The RT-PCR cycle threshold (*C_T_*) values and the time elapsed (in days) between the onset of symptoms and sample collection were recorded. This study received a waiver from the Clinical Research Ethics Committee of the Hospital Clinic of Barcelona.

### Molecular diagnosis.

SARS-CoV-2 molecular diagnosis of NPSs was performed using the Xpress SARS-CoV-2 (GeneXpert, Cepheid, USA) or the TaqPath COVID-19 RT-PCR kit (ThermoFisher Scientific, Waltham, MA).

### Rapid antigen tests.

The following six RATs were evaluated in this study: Coronavirus Ag rapid test cassette (Healgen Scientific, Houston, TX, USA), COVID-19 Ag FIA (Vircell, SD Biosensor Inc., Gyeonggi-do, Republic of Korea), Clinitest rapid COVID-19 antigen test (Siemens, Healthineers, Erlangen, Germany), SARS-CoV-2 rapid antigen test (SD Biosensor, Roche Diagnostics, Basel, Switzerland), Panbio COVID-19 Ag rapid test device (Abbott, Chicago, IL, USA), and SARS-CoV-2 test (MonLab, Barcelona, Spain). Samples in VTM were mixed with the corresponding buffer in a 1:1 proportion, they were loaded into the devices, and the tests were performed and interpreted following the manufacturer’s instructions. After 15 min, the results were read manually except with COVID-19 FIA (Vircell, SD Biosensor) which required an automatic reader. All tests were carried out under biosafety conditions. Samples were thawed once and tested by all six RATs.

### Clinical data and statistical analysis.

The presence or absence of symptoms, the days after the onset of symptoms and sample collection, and the SARS-CoV-2 RT-PCR *C_T_* value were retrieved from the hospital’s medical and laboratory records. In this study, the *C_T_* value corresponding to the N gene was used to compare the sensitivity of the antigen tests in different *C_T_* ranges of the RT-PCR. Rapid antigen diagnostic tests were evaluated for their sensitivity (S) and specificity (Sp) using RT-PCR as a reference. The overall S and Sp were calculated, as well as the performance of the tests depending on the RT-PCR *C_T_* value (<25 and ≥25) and at different time after the onset of symptoms (≤7 and >7 days). Negative predictive value (NPV), positive predictive value (PPV), and accuracy were calculated for different prevalences of the disease. The statistical parameters were calculated using the MedCalc platform (https://www.medcalc.org/calc/diagnostic_test.php, accessed August 2021). The accuracy of the tests (overall probability that a patient is correctly classified) was defined as follows: sensitivity × prevalence + specificity × (1 − prevalence).
