# Supplementary material for: Diagnostic Performance of Six Rapid Antigen Tests for SARS-CoV-2
Source: Microbiol Spectr. 2022 Mar 16;10(2):e02351-21. doi: 10.1128/spectrum.02351-21 (PMC9045193; doi:10.1128/spectrum.02351-21)
Supplement: SUPPLEMENTAL FILE 1 — Supplemental material. Download SPECTRUM02351-21_suppmental_file.pdf, PDF file, 0.04 MB [file spectrum02351-21_suppmental_file.pdf]

		Healgen		VirCell		Siemens		Roche		Abbot		MonLab	
**Disease Prevalence**	**Statistic**	**Value**	**95% CI**	**Value**	**95% CI**	**Value**	**95% CI**	**Value**	**95% CI**	**Value**	**95% CI**	**Value**	**95% CI**
1%	Positive Predictive Value	100.0%		100.0%		100.0%		100.0%		100.0%		100.0%	
	Negative Predictive Value	99.7%	99.5-99.7	99.8%	99.7-99.8	99.8%	99.7-99.9	99.7%	99.6-99.8	99.7%	99.6-99.8	99.6%	99.5-99.7
	Accuracy	99.7%	96.5-100.0	99.8%	96.8-100.0	99.8%	96.6-100.0	99.7%	96.6-100.0	99.7%	96.1-100.0	99.6%	96.5-100.0
5%	Positive Predictive Value	100.0%		100.0%		100.0%		100.0%		100.0%		100.0%	
	Negative Predictive Value	98.3%	97.6-98.7	98.8%	98.2-99.2	98.9%	98.3-99.3	98.6%	98.0-99.1	98.6%	98.0-99.0	98.2%	97.5-98.6
	Accuracy	98.3%	94.3-99.8	98.9%	95.2-99.9	99.0%	95.2-99.9	98.7%	94.8-99.9	98.7%	94.9-99.9	98.2%	94.2-99.7
10%	Positive Predictive Value	100.0%		100.0%		100.0%		100.0%		100.0%		100.0%	
	Negative Predictive Value	96.4%	95.2-97.4	97.5%	96.3-98.3	97.8%	96.5-98.6	97.2%	95.9-98.0	97.1%	95.9-98.0	96.2%	95.0-97.2
	Accuracy	96.7%	91.9-99.0	97.7%	93.4-99.5	97.9%	93.6-99.6	97.4%	92.8-99.4	97.3%	92.9-99.4	96.5%	91.6-98.9
15%	Positive Predictive Value	100.0%		100.0%		100.0%		100.0%		100.0%		100.0%	
	Negative Predictive Value	94.4%	92.5-95.9	96.1%	94.3-97.4	96.5%	94.6-97.7	95.6%	93.7-96.9	95.5%	93.7-96.8	94.1%	92.2-95.6
	Accuracy	95.00%	89.7-98.1	96.58%	91.8-99.0	96.92%	92.1-99.2	96.05%	91.0-98.7	96.01%	91.0-98.7	94.68%	89.3-97.9
20%	Positive Predictive Value	100.00%		100.00%		100.00%		100.00%		100.00%		100.00%	
	Negative Predictive Value	92.31%	89.8-94.3	94.61%	92.1-96.3	95.11%	92.5-96.8	93.83%	91.2-95.9	93.77%	91.2-95.6	91.86%	89.3-93.8
	Accuracy	93.33%	87.5-97.0	95.44%	90.3-98.3	95.89%	90.7-98.6	94.74%	89.3-97.9	94.68%	89.3-97.9	92.91%	87.0-96.7

Supplementary table 1: Simulation of the overall ~~VPP~~, ~~VPN~~ an accuracy of every test according to prevalence of the 1, 5, 10, 15 and 20%.
